# Text messaging interventions to support smoking cessation among hospitalized patients in Brazil: a randomized comparative effectiveness clinical trial

**DOI:** 10.1186/s13104-022-06002-6

**Published:** 2022-03-26

**Authors:** Lígia Menezes do Amaral, Telmo Mota Ronzani, Erica Cruvinel, Kimber Richter, Rafaela de Oliveira Andrade, Isabella Oliveira Lanzieri, Ângela Caroline Dias Albino Destro de Macêdo, Isabel Cristina Gonçalves Leite

**Affiliations:** 1 Clinical Medicine Department, Federal University of Juiz de Fora, Juiz de Fora, Minas Gerais Brazil; 2 Department of Psychology, Federal University of Juiz de Fora, Juiz de Fora, Minas Gerais Brazil; 3 Population Health Department, University of Kansas Medical Center, Kansas City, KS USA; 4 Preventive Medicine and Public Health, University of Kansas Medical Center, Kansas City, KS USA; 5 Present Address: Medical School, Federal University of Juiz de Fora, Juiz de Fora , Minas Gerais Brazil; 6 Medical School, Federal University of Juiz de Fora, Juiz de Fora, Minas Gerais Brazil; 7 Collective Health Department, Federal University of Juiz de Fora, Juiz de Fora, Minas Gerais Brazil

**Keywords:** Patient discharge, Randomized trial, Smoking cessation, Text messaging

## Abstract

**Objective:**

A clinical trial carried out in patients hospitalized for clinical and surgical conditions. This study evaluated the effectiveness of text messaging interventions (TM) versus telephone counseling (TC) to promote smoking cessation among hospitalized smokers in a middle-income country. Seven-day abstinence was measured during follow-up phone calls one month after discharge. The comparative cost of the two interventions considered the cost of calls, time spent on phone calls and sending SMS and cost of the professional involved in the approaches.

**Results:**

Past 7-day tobacco abstinence was not statistically different between groups (30.5% in TM group and 26% in TC, p = 0.318). Costs were significantly lower in the TM group (U$9.28 × U$19.45- p < 0,001). Continuous abstinence was reported by 26% of TM participants and 24.5% of TC participants (p = 0.730). In the 3-month follow-up, 7-day abstinence was 23% in the TMI and 27% in the TC (p = 0.356) group. Continuous abstinence was reported by 20% of TM participants and 24% of TC participants (p = 0.334).

*Trial registration*: ClinicalTrials.gov ID: NCT03237949 Registred on: 30th May 2017.

**Supplementary Information:**

The online version contains supplementary material available at 10.1186/s13104-022-06002-6.

## Introduction

Smoking is the leading cause of premature death worldwide [1, 2]. During hospital stays, when they were hospitalized for diagnosis and treatment of clinical and surgical conditions, patients must abstain from smoking, and they are particularly accessible and interested in receiving advice to quit [3–7]. Increased access to phones, cell phones, the internet, and the emergence of quitlines have made these strategies attractive vehicles for novel health interventions [8–15].

Brazil has one of the world’s most successful tobacco control programs, leading to a significant reduction in the prevalence of smoking in the last three decades (from more than 40% to less than 10% of the population). Brazil implemented numerous tobacco control policies including smoke-free air laws, marketing restrictions, graphic health warnings on cigarette packaging, national smoking cessation campaigns through the mass media, cigarette price increases and a national smoking cessation treatment program [16–19]. However, only a few hospitals in Brazil actually have a protocol to address smoking cessation with their patients [20–23].

Text messaging shows strong potential to extend care to hospitalized smokers in Brazil during the post-discharge period [3]. Text message interventions are effective for smoking cessation [24–26] and have a wide reach and low cost [24]. Most Brazilians- 86% of the population aged 10 years or over- are mobile phone users [27].

The present study is the first definitive randomized clinical trial to evaluate SMS for the post-discharge follow-up of smokers in Brazil. However, most smokers do not receive smoking cessation treatment when trying to quit [3]. Different from other countries, there is limited research investigating SMS to support smoking cessation among post-discharge patients in Brazil.

The objective of the present study was to evaluate the effectiveness and costs of a post-discharge text messaging (TM) versus telephone counseling (TC), for supporting cessation among hospitalized smokers in Brazil.

## Main text

### Methods

#### Design

The design a 1:1 ratio two-arm randomized controlled clinical trial of non-inferiority conducted with hospitalized smokers. The study was approved by the Hospital Ethics Committee Review Board/process number 2.868.112. The trial is registered in the Clinical Trials Registry (NCT03237949) and the Brazilian Clinical Trials Registry (RBR-8mgc3h). The study adheres to the CONSORT Guidelines.

#### Setting and participants

This study was conducted at a university hospital located in the city of Juiz de Fora in the southeastern part of Brazil. Smoking is prohibited in the hospital (tobacco-free campus) and patients had to remain abstinent throughout their stay. Patients were recruited from all units of a 159-bed public university hospital. Participants were involved in an initial pre-screening process using the Electronic Medical Record System (EMR) and then answered an in-person screening survey administered by the researcher team. In the first interview during hospitalization, eligible participants were consented and answered the baseline survey at bedside the day after hospitalization.

During the hospital stay and following discharge, the Interdisciplinary Center for Research and Intervention in Tobacco (CIPIT) provides tobacco treatment to all patients [28]. All patients also received a motivational approach during hospitalization and after discharge according to their allocation group.

Hospitalized smokers were eligible for inclusion on the study if they were aged ≥ 18 years, had smoked cigarettes within the past 30 days, had a cell phone, had received at least one text message in the past year. Participants were excluded if they were admitted to the intensive care, isolation rooms, were physically or cognitively unable to participate, or were incarcerated.

With 200 participants in each group, the study had 80% statistical power at an alpha of 0.05 to detect a 10% difference between groups in the proportion of participants who would quit smoking in the third month [3].

#### Randomization

A random allocation sequence was computer generated by the CDC Epi InfoTM7 platform. Participants were randomly assigned after the discharge, and the allocation order followed the discharge order (time and date).

### Interventions

#### Text messaging

Patients randomized to TM received up to 30 text messages for 8–15 days after discharge. The number of messages sent after discharge was determined by the degree of motivation to maintain abstinence, reported by the patient while still hospitalized. The frequency and content of the messages varied depending on the participants’ motivation to quit. Patients abstinent or preparing to quit in the next 14 days received two messages/day over the course of 15 days. Patients not ready to quit received two messages/day over the course of 8 days following hospitalization.

The format and design of the text messages followed methods used in the pilot study [3]. Some messages were common to all patients and others were personalized based on information obtained in interviews during hospitalization. Bandura’s self-efficacy theory to design the content of text messages was used [29]. The study did not assess abstinence in the last 15 days but we used the intention to quit (or remain abstinent) in the next 30 days, collected in the baseline assessment, to customize the text message content. Participants abstinent or preparing to quit in the next 30 days received two messages per day for 15 days. Text messages were sent manually by a cell phone. Messaging was unidirectional-participants were unable to reply to messages.

#### Telephone counseling

In TC, participants received one weekly phone call for 4 weeks. Four attempts were made, on different days and times of the day, per week for each participant. Telephone counseling lasted approximately 15 to 30 min. The counseling approach addressed motivation, confidence, quitting history, environmental factors, trigger situations, coping strategies, medication use, relapse prevention and setting a quit date. This is the standard treatment given to all discharged patients, except in the intervention group (TM) during this study. The approaches were based on concepts of motivational interviewing and cognitive behavioral therapy. Each telephone counseling was designed to help patients develop an individualized plan to quit smoking or to remain abstinent. The approach was based on Motivational Interviewing (quote) and addressed behavioral and cognitive issues, including motivation, confidence, quitting history, environmental factors, trigger situations, coping strategies, medication use, relapse prevention and setting a quit date [30].

These approaches were based on previou pragmatic tobacco treatment research [3].

#### Measures

Measures included demographics and social class distribution [30]. The nicotine dependence was evaluated via Fagerström Test [32]. Withdrawal symptoms during hospitalization [33], tobacco use characteristics, readiness to quit [34], and nicotine replacement therapy during hospitalization were assessed. The Patient Health Questionnaire –4 (PHQ-4) and the Alcohol Use Disorder Identification Test (AUDIT-C) were used [35, 36].

The main outcome measure was self-reported 7 day point prevalence abstinence at 1 month post-randomization (“Did you smoke even a single puff in the last 7 days?”). Secondary outcomes included self-reported 30 days continued abstinence at 1-month post-randomization (“Did you smoke even a single puff in the last 30 days?), and biochemically verified abstinence at 3 months post-discharge. Exhaled carbon monoxide of ≤ 10 ppm was the cutoff for verification of abstinence [37].

To calculate costs, we analyzed the average time spent on interventions, the minute value of each intervention per participant, the amount paid to the telephone company, and the cost per minute worked by the healthcare professional, based on the federal employees’ salary scale. Costs were calculated in Brazilian Real and converted into dollars on January 7. 2020 (1 dollar = 4.08 Brazilian Real).

#### Analyses

Research Electronic Data Capture (REDCap) was used to enter the data. After descriptive statistics, the comparison of categorical variables was performed by chi-square and, for continuous variables with normal distribution, t-tests for independent samples. Nonparametric distribution variables were analyzed by Mann–Whitney. Subjects lost to follow-up were counted as smokers (intention to treat analysis—ITT).

A comparative assessment of intervention costs was performed using cost minimization, used to measure the cost difference between alternative interventions, when it is assumed that both have the same effectiveness [38].

### Results

Participants were recruited from May 2017 to January 2019. Of 629 individuals identified as smokers, after evaluating exclusion criteria, 400 participants were randomized and allocated to the study groups. Some interruptions occurred due to the worsening of the medical condition of the patients or for the performance of complementary exams at the time of the approach (Figure S1).

### Participant characteristics

Randomization led to similar groups for all baseline characteristics, except for the age of tobacco initiation (Table 1).

**Table 1:** Baseline characteristics of study participants by treatment group

Variables	Standard care (control)	Sustained care (intervention)	p
	**M (SD)**	**M (SD)**	
Age (years)	45.97 (12.58)	45.45 (12.92)	0.701
Age at smoking initiation	18.70 (16.35)	16.09 (7.40)	**0.001**
	**Md (IR)**	**Md (IR)**	
Importance of quitting (0–10)	10 (0)	10 (0)	0.844
Confidence to quit (0–10)	7 (5)	8 (5)	0.273
Withdrawal scale (0–4)	3 (4)	3 (4)	0.303
	**N (%)**	**N (%)**	
Male	102 (51.3)	94 (47.2)	0.422
Ethnoracial group (self-declared)			0.511
White	67 (36.2)	62 (33.0)	
Black, grayish-brown/indigenous	118 (63.8)	126 (67.0)	
Education level			0.201
0–4 years	5 (2.6)	1 (0.5)	
5–8 years	78 (40.4)	75 (38.1)	
More than 9 years	110 (57)	121 (61.4)	
Married or whit a partner	61 (33)	58 (32.2)	0.878
SES^1^—average household income in dollars/month^2^			0.228
SES A (USD 5.058)	1 (0.5)	0.0 (0.0)	
SES B1(USD 2.241)	2 (1.0)	1.0 (0.5)	
SES B2 (USD 1.175)	43 (21.1)	30 (15.0)	
SES C1 (USD 655)	112 (56.3)	110 (55.0)	
SES C2 (USD 393)	42 (21.1)	58 (29.0)	
SES D + E (USD 186)	0 (0.0)	1.0 (0.5)	
Cigarettes/day			0.165
< 10	81 (40.5)	94 (47.3)	
11–20	72 (36.0)	83 (41.7)	
> 21	47 (23.5)	22 (11.0)	
Time to first cigarette of the day			0.264
After 60 min	34 (17.3)	52 (26.4)	
Between 31 and 60 min	24 (12.2)	19 (9.6)	
Between 6 and 30 min	61 (31.0)	43 (21.8)	
The first 5 min	78 (39.5)	83 (42.2)	
Nicotine dependence^3^ ≥ 5	168 (84.0)	179 (89.5)	0.105
Quit attempts in past year	69 (34.5)	87 (43.5)	0.065
Life use quit medication			0.742
NRT	32 (16.1)	34 (16.9)	
Bupropion	15 (4.6)	15 (7.5)	
Champix or Varenicline	2 (1.0)	0 (0.0)	
Commitment to quitting			0.711
Plan to stay quit	118 (62.8)	125 (64.4)	
Plan to try to stay quit	25 (13.3)	27 (13.9)	
Plan to reduce smoking	34 (18.1)	28 (14.4)	
Plan not to quit	11 (5.9)	14 (7.2)	
Current depressive symptoms^4^	88 (56.1)	88 (55.3)	0.900
Current anxiety symptoms^5^	96 (72.2)	98 (76.6)	0.418
Mild to Severe Risk of alcohol abuse^6^	133 (66.5)	123 (61.5)	0.298
NRT during hospitalization	188 (94.0)	190 (95.0)	0.661
Any smoking-cessation treatment	23 (13.9)	26 (20.5)	0.133
Any smoking-cessation counseling	57 (34.8)	37 (29.6)	0.354
Interest in receiving medication	170 (89.9)	171 (91.9)	0.503

*M* Mean, *SD* Standard deviation, *Md* Median, *IR* Interquartile range, *SES* Social economic stratum, *NRT* Nicotine replacement therapy

^1^Assessed via Brazilian criteria and social class distribution (ABEP 2016)

^2^1 real = 4.129 dollars, december 09, 2019

^3^Assessed via Fagerström test for nicotine dependence

^4,5^Assessed via patient health questionnaire 4 item (PHQ-4)

^6^Assessed via alcohol use disorder identification test (AUDIT-C)

The proportion of participants reached for follow-up was 73.25% (n = 293) at 30 days post-discharge and 66% (n = 264) at 90 days post-discharge.

### Abstinence at 1 and 3 months after discharge

Self-reported, 7 day point prevalence abstinence rates were not statistically different between groups at 1 month post-discharge using an ITT analysis (p = 0.318). Similarly, 30 day continuous abstinence was not significantly different between groups (p = 0.730), however the number of cigarettes per day smoked was fewer in the TM group (p < 0,036). Quit rates were also not significantly different at 3-month follow-up (Table 2).

**Table 2:** Abstinence at follow up post-discharge by treatment group

Variables	Sustained care (Intervention) N (%)	Standard care (Control) N (%)	p
Follow-up 30 (missing = smoking)^1^			
Abstinences for the past 7 days	61 (30.5)	52 (26.0)	0.318
Abstinences for the past 30 days	52 (26.0)	49 (24.5)	0.730
Follow-up 90 (missing = smoking)			
Abstinences for the past 7 days	46 (23.0)	54 (27.0)	0.356
Abstinences for the past 90 days	40 (20.0)	48 (24.0)	0.334
Abstinences verified by measurement of exhaled carbon monoxide^2^	20 (95.2) n = 21	8 (80.0) n = 10	0.160
	**M (SD)**	**M (SD)**	
Number of cigarettes per day in non-abstinent participants at follow-up 30	9.99 (15.033)	13.91 (14.859)	**0.036**
Number of cigarettes per day in non-abstinent participants at follow-up 90	10.04 (14.997)	12.51 (12.421)	0.131

*M* Mean, *SD* Standard deviation

^1^Subjects lost to follow-up were counted as smokers (intention to treat analysis—ITT)

^2^Participants were characterized as abstinent if their results were ≤ 10 ppm

### Cost analysis

Costs were significantly lower in the TM group compared to the TC group. Cost results can be seen in Table 3.

**Table 3:** Cost analysis by strategies of counseling post-discharge

Analysis	Text messages interventions	Telephone calls	p
Quantity per patient	14 a 30	4	
Monthly cost of telephone company	R$ 22.50 U$5.51	R$ 69.90 U$17.13	***p*** **< 0,001**
Average time	1 message- 0.5 min	1 call- 4.7 min	
Average number of retries per approach	1	3.15	
Time spent on unsuccessful attempts per patient	0 min	8 min	
Health worker’s minute value ^1^	R$ 0.77 U$ 0.18	R$ 0.77 U$ 0.18	
Average time per patient	7/15 minutes^2^	26.8 minutes^3^	
Total cost per patient	R$ 5.39 / R$ 11.55 U$ 1.32/2.83	R$ 20.63 U$ 5.06	***p *< 0.001**
Cost total per group (200 participants)	R$ 2.310.00	R$ 4.126.00	
Cost per abstinent participant in last 7 days with 30 days of follow-up	(61 participants) R$ 37.87 U$9.28	(52 participants) R$ 79.34 U$19,45	***p *< 0.001**

^1^According to Table of Salaries of Civil Federal Civil Servants of Jan 2019 –Education Technicians Category E (undergraduate level)

^2^Referring to 15 messages for the least motivated group and 30 messages for the group motivated for cessation

^3^Sum of call times completed, and average spent on unsuccessful attempts per patient

### Discussion

This was the first fully-powered study to compare the effectiveness of text messaging versus telephone counseling for post-discharge smoking cessation treatment. Both led to a high prevalence of self-reported smoking abstinence. Cost analysis found that text messaging intervention was half as expensive as phone calls.

A recent meta-analysis included 26 clinical trials and concluded that there is moderate evidence that text messages increase cessation rates by approximately 50% when compared to support for smoking cessation [26].

In the last decades, there has been a great advance in the use of communication technologies in health care. This phenomenon contributed to the emergence of innovative health behavior change interventions [24] and several strategies have been studied to help hospitalized smokers to quit [11, 12, 15].

Behavior change interventions sent by text messages are becoming increasingly popular, the possibility of reaching many people when performing interventions without personal contact reduces costs and allows access to people who are reluctant to have direct contact [24, 25, 39, 40].

Text messages have been used in Brazil for approximately 30 years, but the strategy, despite offering great advantages in the health area, is still little explored for this purpose. Despite the Brazilian tobacco control program reaching a significant number of people, through actions of the Unified Health System (SUS), the country’s free public health system, communication technologies such as SMS are still not used by the program. Text messaging strategies are promising especially for low/middle-income countries where proactive telephone counseling is not available for free and quitline services are not structured. Future studies should address the effectiveness of automated messaging systems, evaluate ways to promote better interactivity with the participants, and determine the intensity of the approach to deliver the best results.

## Limitations

Loss of follow up is an important limitation of longitudinal studies. In this study, there were significantly fewer reached for follow up in the standard care (TC) group than in the TM group. Other limitations are the loss of interactivity due to the lack of actions aimed at possible participants’ responses to messages (unidirectional messaging) and the unavailability of an automated messaging system.

## Supplementary Information

**Additional file 1: Figure S1.** Participant flow diagram.

## Data Availability

The datasets used and/or analysed during the current study available from the corresponding author on reasonable request.

## Abbreviations

TM: Text messaging
TC: Telephone counseling
CIPIT: Interdisciplinary center for research and intervention in tobacco
CDC: Center disease control
PHQ-4: Patient health questionnaire–4
AUDIT-C: Alcohol use disorder identification test
REDCap: Research electronic data capture
ITT: Intention to treat analysis
M: Mean
SD: Standard deviation
Md: Median
IR: Interquartile range
SES: Social economic stratum
NRT: Nicotine replacement therapy
ppm: Particles per million
