# Supplementary figures and images for: Text messaging interventions to support smoking cessation among hospitalized patients in Brazil: a randomized comparative effectiveness clinical trial
Source: BMC Res Notes. 2022 Mar 26;15:119. doi: 10.1186/s13104-022-06002-6 (PMC8962029; doi:10.1186/s13104-022-06002-6)

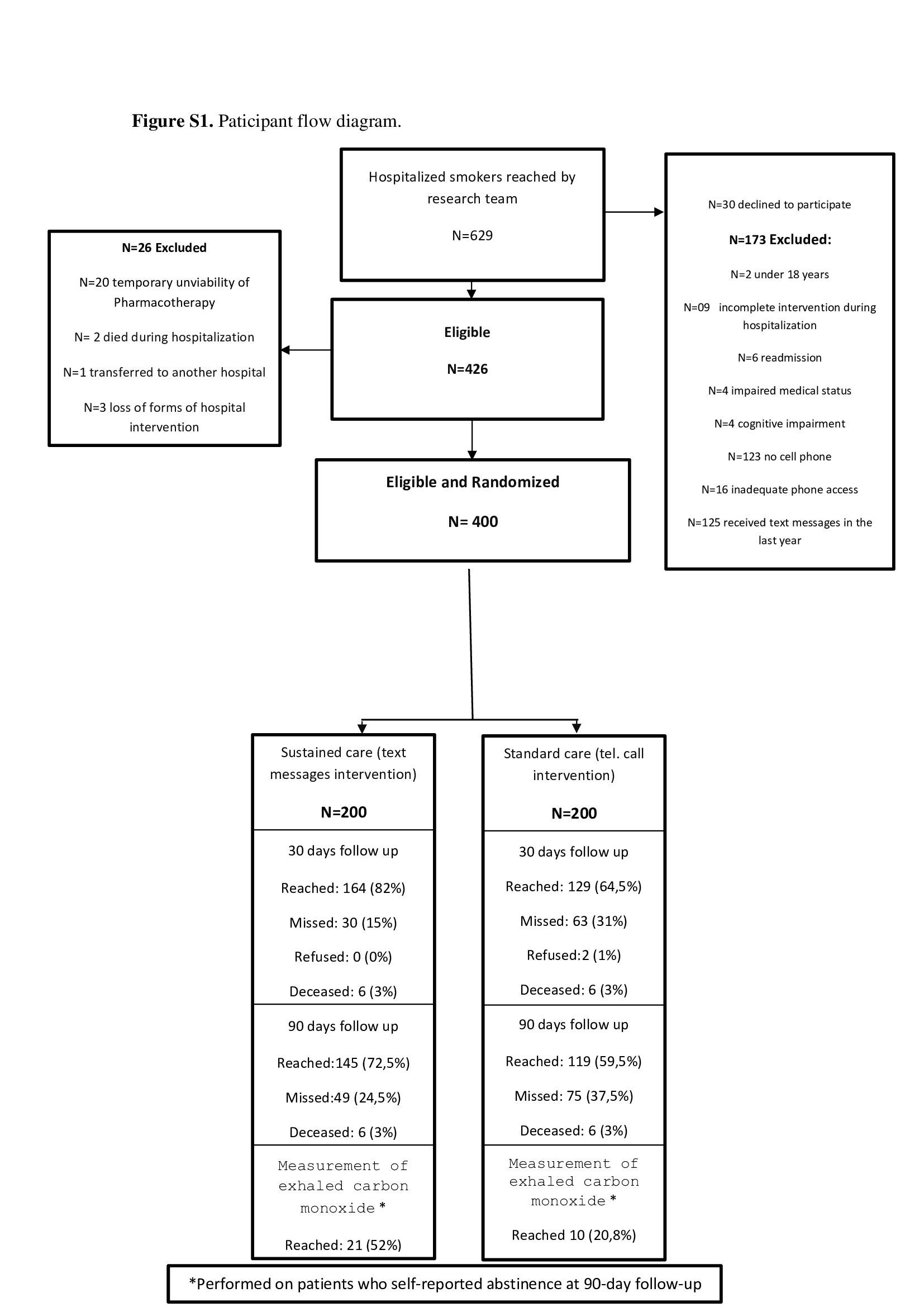

Supplement: Supplementary file 1 — Additional file 1: Figure S1. Participant flow diagram. [file 13104_2022_6002_MOESM1_ESM.jpg]
